# Distribution of Galepsus spp. in Southern Africa and Life History of *Galepsus lenticularis* (Mantodea: Tarachodidae)

**DOI:** 10.3390/insects11020119

**Published:** 2020-02-11

**Authors:** Bianca Greyvenstein, Hannalene Du Plessis, Nicolas Moulin, Johnnie Van den Berg

**Affiliations:** 1 Unit for Environmental Sciences and Management, North-West University, Potchefstroom 2520, South Africa; hannalene.duplessis@nwu.ac.za (H.D.P.); johnnie.vandenberg@nwu.ac.za (J.V.d.B.); 2 Institut Systématique, Evolution, Biodiversité (ISYEB), Muséum national d’Histoire naturelle, 75231 Paris Cedex 05, France; nmentomo@gmail.com

**Keywords:** biology, competition, distribution, mantis, resources

## Abstract

*Galepsus* Stäl is a genus within the Mantodea and has hardly been studied in Africa. The distribution of the *Galepsus* genus in Southern Africa was established, based on insect collection records, and the biology of *Galepsus* (*Lygdamia*) *lenticularis* Saussure, was studied. In Southern Africa, 11 species of *Galepsus* were recorded. The first record of *Galepsus* (*Onychogalepsus*) *centralis* Beier, in South Africa was recorded during this study. The mean number of eggs per ootheca was 49.8 (±21.1) and unfertilized oothecae were significantly shorter and contained fewer eggs than hatched and unhatched oothecae, suggesting that females might invest fewer resources into production of oothecae that will not produce prodigy. No parthenogenesis was observed during this study. Although the mean duration of the male and female nymphal stages were similar, longevity of adult females (91.2 ± 35.0 days) was three times longer than that of males (26.3 ± 15.4 days). This phenomenon as well as the long period (20 ± 14.1 days) between oviposition of different oothecae, and duration of the incubation period (20.25 ± 6.3 days) suggests a survival strategy to reduce competition between siblings. Total longevity of males (166.9 ± 38.8) and females (252.9 ± 54.2) differed significantly. This study provides information on the distribution of *Galepsus* spp. in Southern Africa and describes the biology of *G. lenticularis* under captive breeding conditions, and contributes to the understanding of various biological aspects of *G. lenticularis* which has never been studied before.

## 1. Introduction

The Mantodea is a small arthropod order and, because they are thermophilic, their distribution linked largely to tropic and subtropical regions. Mantid distribution is limited to tropical and subtropical regions between the 45–46 degree latitudes [1]. It is estimated that there are approximately 2600 mantid species globally [2,3,4,5,6]. The Mantidae, with approximately 1000 species, is the largest of the 21 families in the Mantodea [6,7,8,9]. The Tarachodidae family has 253 species and is well presented in Southern Africa [5,6,10,11].

South Africa is poorly represented with regard to knowledge of the distribution and species richness of the Mantodea [11]. Africa is one of the continents with the greatest number of Mantodea species, thus South Africa could have a significant number of species [5,12]. However, only approximately 197 species of mantids in 11 families have been recorded in South Africa [11], slightly more than the 180 mantid species reported earlier [10]. The only surveys of Mantodea in South Africa were done by Kaltenbach from 1996 to 1998 [10,13] and a survey in 2005 in three areas (Cape floristic region, Richards Bay in the Kwa-Zulu Natal province and the Kruger National Park in the Mpumalanga province). The latter survey was done by the Mantodea Project which is an affiliation of the Cleveland Museum of Natural History in Ohio, USA [14]. Due to the lack of knowledge regarding Mantodea, it is possible that there could be significantly more species, especially considering that South Africa’s endemism rate for invertebrates is estimated at 70% [15]. Scientific reports on Mantodea biology and distribution are limited throughout the world, and in South Africa, largely absent.

*Galepsus* is a genus in the Tarachodidae family and there are four subgenera and 67 valid species within this genus [6], some of which have only recently been described, i.e., *G.* (*Syngalepsus*) *dudleyi* Moulin 2018 and *G.* (*Syngalepsus*) *bucheti* Moulin 2018 [16]. *Galepsus* is one of the genera that require global revision, especially those from under-collected regions such as Sub-Saharan Africa [17]. The distribution of *Galepsus* is estimated to be mostly in Sub-Saharan Africa, Fiji and the island of the Comoros [5].

*Galepsus* spp. seem to be common in the grassland biome of South Africa. A total of 202 *Galepsus* individuals were recorded in one short term biodiversity survey (32,400 m^2^) which was done in agricultural rangeland and crop fields in the Highveld Grassland Biome in South Africa [18], while another study recorded 72 *Galepsus* individuals in the same region (2400 m^2^) [19]. *Galepsus* spp. was also commonly collected in a study of arthropod diversity in ruderal green space within urban areas in the Grassland biome of South Africa [18]. However, few studies mention *Galepsus* (*Lygdamia*) *lenticularis* Saussure 1872. However, it the distribution of the species was reported to be throughout Southern Africa [5], while [10] listed only 10 distribution records of this species in the region. The lack of knowledge and need for research on *G. lenticularis* was also highlighted on a taxonomic website dedicated to this group, i.e., Mantodea Species file [6]. Basic biological and distribution information regarding *Galepsus* spp. in general and *G. lenticularis* in particular would contribute to information on this arthropod group and species in the Grassland and Savanna biome of Southern Africa. The aim of this study was to compile distribution maps of *Galepsus* spp. in Southern Africa and to study the biology *G. lenticularis*.

## 2. Materials and Methods

### 2.1. Species Distribution Database

Distribution records of *Galepsus* spp. were collected during visits to the following institutions that host insect collections in South Africa: Ditsong Museum of Natural History (Pretoria), Agricultural Research Council (Biosystematics Division in Pretoria), National Museum (Bloemfontein), Albany Museum (Grahamstown), Rhodes University (Grahamstown), Durban Natural Science Museum, Iziko South African Museum (Cape Town), and KwaZulu-Natal Museum (Pietermaritzburg). Most specimens in these collections where previously identified by visiting taxonomists while many were sent for identification to the Vienna Museum in Germany, the University of Drexel in Philadelphia, USA, the Muséum national d’Histoire naturelle (MNHN) in Paris, France and the research collection of Nicolas Moulin in Montérolier, France. Southern Africa in the context of this paper includes the following countries: Angola, Botswana Lesotho, Mozambique, Namibia, South Africa, Eswatini, Zimbabwe, and Zambia. This is due to the lack of specimen records from other African countries in the museum collections in South Africa, other African countries were not included. *Galepsus* specimens and distribution labels where photographed (Canon EOS D1300, Canon, Tokyo, Japan), digitized and a database was compiled. This database contains the following information for each specimen record: Genus and species name (to the available level of identification), collector’s details and collection date where available, and the geo-referenced locality. A website (http://Mantodea.speciesfile.org) and literature were used to determine the current nomenclature within the genus. All locality data was georeferenced using the principles suggested [20] and all coordinates were converted from degrees, minutes, and seconds (DMS) to decimal degrees (DD) with the use of the website (gps-coordinates.net). DD were used for developing the distribution maps for *Galepsus* species in Southern Africa by means of GIS software (ArcMaps, Version 10.6.1). 

### 2.2. Rearing and Biology of Galepsus Lenticularis

Individuals were collected in the Grassland biome in the North-West and Free State provinces of South Africa during the summer of 2016/2017. These field-collected individuals were in the adult phase and were allowed to mate and lay eggs in order to get sufficient numbers of individuals to use for the captive breeding and biological studies. A sub-sample of the field-collected specimens was identified by Nicolas Moulin, honorary associate to MNHN.

For breeding purposes, pairs of male and females were placed in glass containers. One-liter glass containers were used to ensure that ample space was available for the male to increase the chances of a successful escape after mating. To further limit the likelihood that females would cannibalize the males during or after mating, ample food was provided before the male was introduced into the breeding container. After copulation concluded, the male was removed from the breeding container. 

The terrarium (15 cm × 10 cm × 20 cm) in which females were kept after mating were checked daily for the presence of oothecae that was laid overnight. Oothecae were removed and put into small (5 cm diameter and 5 cm high) containers inside a desiccator with potassium hydroxide (KOH) to ensure a humidity level of 68% ± 5% within the closed desiccator [21]. The desiccator was kept in an insect rearing room at a temperature of 27 ± 1 °C until nymphs emerged from the oothecae. 

Rearing of emerged nymphs was done under controlled conditions. Each specimen was placed in a plastic honey jar (7 cm diameter and 15 cm high) with three holes (each 2 cm in diameter) covered with gauze to allow air flow, hereafter referred to as terrariums. Thin branches (5 mm × 10 cm) were placed inside each jar for climbing and hanging purposes, especially during molts. Food was provided every second day and a fine water mist was sprayed into each container. Live aphids (10) (*Brevicoryne* spp.) (Hemiptera: Aphididae) were used to feed the first and second instars of *Galepsus* while live crickets (2) (*Acheta* sp. Orthoptera: Gryllidae) of different sizes (nymphal instars, i.e., pinheads) were used to feed the nymphs from the 3^rd^ instar onwards. Instances where previous food was not consumed, no additional food was added to prevent over feeding. After molting to the second instar, nymphs were removed from the communal terrarium and placed in separate terrariums to prevent cannibalism. Nymphs were reared until adulthood after which males and females were identified. Observations continued until all individuals died. 

**Figure 1:**
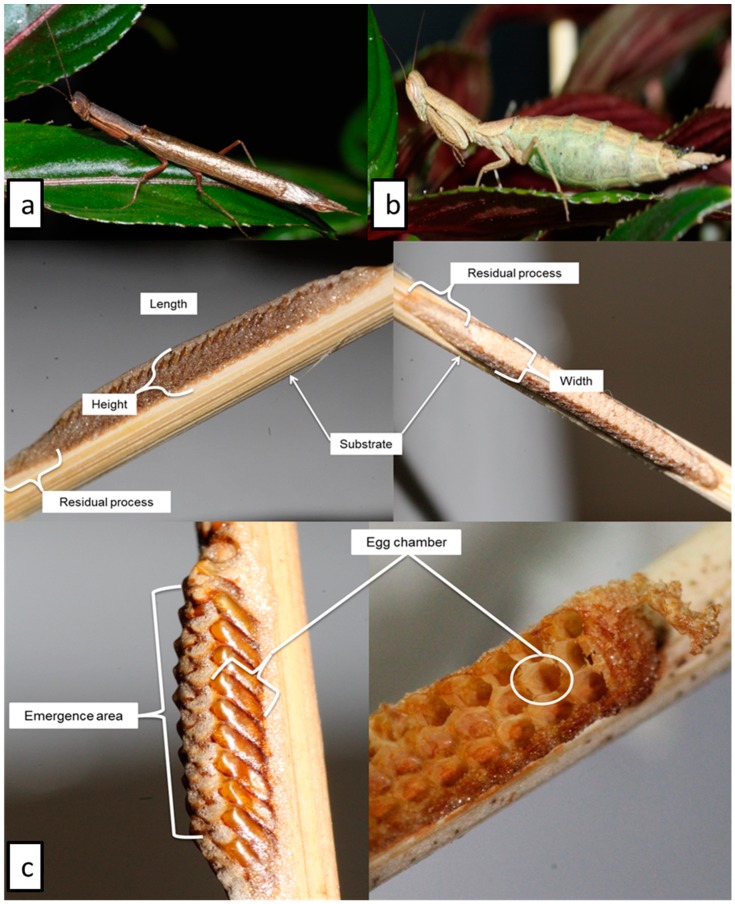
*Galepsus lenticularis* male (**a**) and female (**b**), and general morphology (**c**) of the oothecae, indicating different parameters and areas of interest as suggested by Brannoch [23].

After the final molt, each individual was sexed. This was by means of counting the number of abdominal segments and the presence of wings. *Galepsus lenticularis* females have only reduced wing buds and six abdominal segments while males have eight segments and fully developed wings [7,22,23] (Figure 1a,b).

The following life history parameters were recorded during this study: Size of oothecae, number of egg chambers inside hatched and unhatched oothecae, numbers of days between molts and survival rate to the adult phase. The mean number of days between molts and days to adulthood were calculated separately for males and females. The data recorded and discussed in this paper were recorded for 48 individuals (30 males and 18 females) that completed their life cycles. The mean duration of male and female life cycles was calculated and fertility, hatching, and survival rate determined. A distinction was also made between different types of oothecae, i.e., hatched and unfertilized (field collected as well as from laboratory reared females), and unhatched (field-collected batches laid by females of which the mating status was not known).

The length, width, and height of each ootheca were recorded, based on descriptions (Figure 1c) [23]. The length of the ootheca was measured from the first egg chamber to the last egg chamber and did not include the residual process [23].

### 2.3. Data Analysis

The descriptive statistics (Means and Standard Error) and the statistical analyses and of the developmental parameters of *G. lenticularis* were done using Statistica Version 13.3 [24]. Analyses of Variance (ANOVA) were used to determine if differences existed between the size (length, width, and height) and the number of egg chambers contained by each of the three types of oothecae (i.e., hatched, unfertilized, and unhatched). The mean numbers of days between molts, adult longevity and mean number of days to reach adulthood were also analyzed by means of ANOVAs and compared between the sexes. All significant differences were further analyzed using a post hoc Tukey honest significant difference (HSD) test. 

## 3. Results

### 3.1. Distribution of Galepsus

Distribution records reported in this paper were compiled from records that are available in seven South African institutions that host curated arthropod collections and were identified by a taxonomist with expertise in Afro-tropical Mantodea and are based in Europe (3rd author of this paper). The results presented in this paper should be viewed in this context, since no specimen records were included beyond those residing in South Africa. 

**Figure 2:**
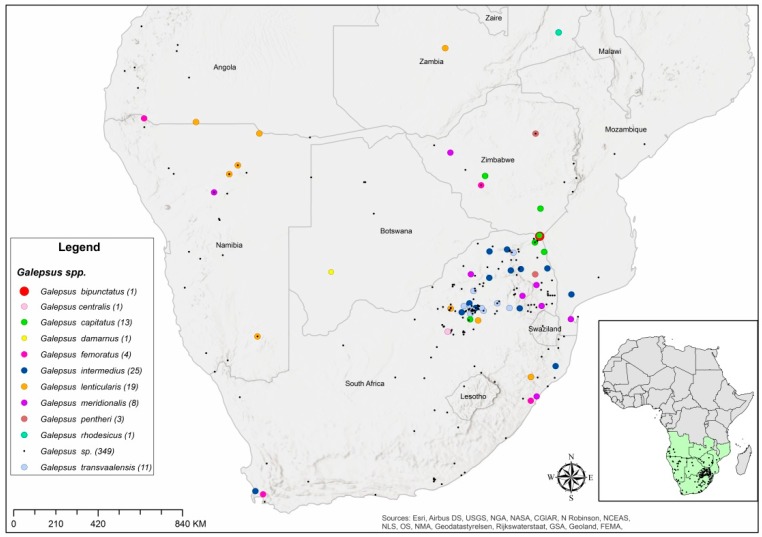
Distribution records of the eleven *Galepsus* species that occur in Southern Africa. Numbers in brackets indicate the number of individual records per species of *Galepsus*. The smaller map of Africa indicates the geographic region defined as Southern Africa in the context of this paper.

A total of 435 specimens of *Galepsus* spp. collected between 1897 and 2016 were recorded in museum collections in South Africa. Most records (71 of 81 specimens) originating from beyond the borders of South Africa (93.7%) were collected between 1897 and 1974, with the majority (50.6%) of records (36 specimens) being collected between 1963 and 1973. Only 20% (89 specimens) of all specimens in South African museums were identified to species level (Table 1). The distribution records also included several other Southern African countries: Angola, Botswana, Eswatini, Lesotho, Mozambique, Namibia, Zambia, and Zimbabwe (Figure 2). 

The sites at which *Galepsus* species were collected are scattered throughout South Africa and the neighbouring countries which cumulatively constitute Southern Africa (Figure 2). Eight of the ten *Galepsus* spp. was only collected in South Africa. Only a single specimen each of *Galepsus* (*Onychogalepsus*) *damaranus* Giglio-Tos, 1911 and *G.* (*Onychogalepsus*) *rhodesicus* Beier, 1954, from Botswana and Zambia respectively, exists for these two species (Table 2). A single record of an unidentified *Galepsus* sp. was recorded in Lesotho as well as in Eswatini. 

The oldest collection records of *Galepsus* spp. in Southern Africa dates back to 1897. These specimens are held at the Iziko South African Museum in Cape Town. One specimen was identified as *G.* (*Onychogalepsus*) *femoratus* Giglio-Tos 1911 while the other is yet to be identified to species level. Information on the date of collection of 386 of the available records indicated that most of the *Galepsus* specimens were collected between 1993 and 2004. This number constitutes 20% of the total number of records of this genus in Southern Africa over the past 120 years.

*Galepsus* (*Onychogalepsus*) *femoratus* Giglio-Tos 1911 and *G.* (*Onychogalepsus*) *intermedius* Werner 1907 were each recorded in three countries, including South Africa, despite *G. intermedius* being the most abundant species with 25 distribution records (Table 2). While *G. lenticularis* however was not the most abundant in the museum collections, it was the most prevalent since it was recorded from Angola, Mozambique, Namibia South Africa, and Zimbabwe. South Africa is thus the only country in Southern Africa where all three *Galepsus* subgenera have been recorded (Table 2). 

*Galepsus* (*Onychogalepsus*) *transvaalensis* Beier 1954 and *G.* (*Syngalepsus*) *bipunctatus* Beier 1931 were recorded only within South Africa’s borders, with 11 records of *G. transvaalensis* from the Gauteng province and a single record of *G. bipunctatus* at Pafuri in the Kruger National Park, close to the border of South Africa and Zimbabwe (Figure 2). All recorded species of *Galepsus* belong to the subgenus *Onychogalepsus* except for *G. bipunctatus* which is the only representative of the subgenus *Syngalepsus* and *G. lenticularis* which is the only representative of the subgenus *Lygdamia*. *Galepsus* (*Onychogalepsus*) *capitatus* Saussure 1869 and *G.* (*Onychogalepsus*) *pentheri* Giglio-Tos 1911 were recorded only in South Africa (two records) and Zimbabwe (one record). 

During collections of specimens (2016–2018) for the breeding and biology of G. *lenticularis,* one record of *G. centralis* Beier, 1957 in the subgenus *Onychogalepsus* was collected in Potchefstroom in the North West province of South Africa. It should be noted that this is the only record of *G.* (*Onychogalepsus*) *centralis* Beier 1957 in South Africa (Table 1 and Figure 2).

**Figure 3:**
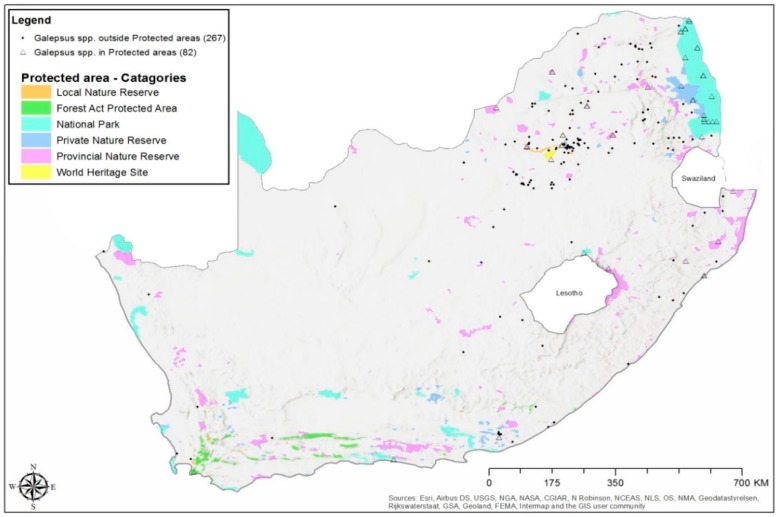
Distribution records of unidentified *Galepsus* spp. collected in protected and non-protected areas of South Africa.

**Figure 4:**
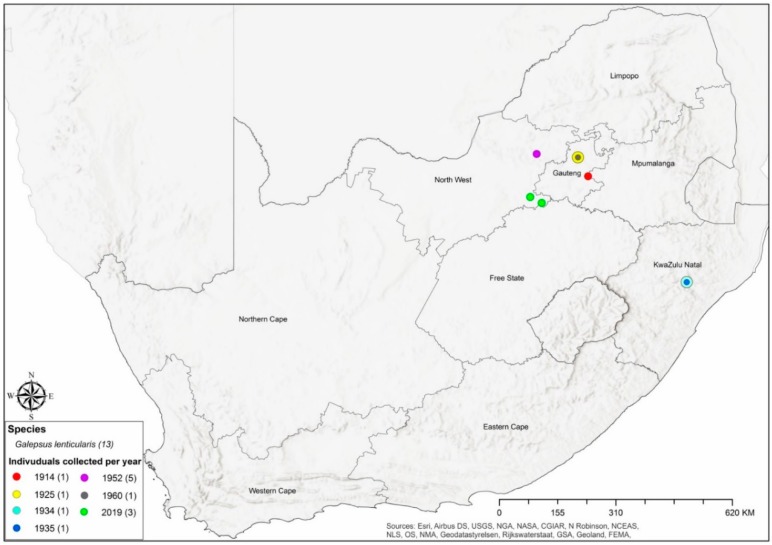
Distribution of *Galepsus lenticularis* based on current and historic records. The sizes of points on the map differs to enable distinguishing of overlapping points.

Most of the *Galepsus* specimens were collected outside of the various different protected areas in South Africa (Figure 3). A total of 267 (76%) of the specimens were collected outside protected areas while Provincial nature reserves and National parks respectively contributed 36 (44%) and 31 (38%) specimen records. The distribution based on records of *Galepsus lenticularis* is depicted in Figure 4.

### 3.2. Biology of Galepsus lenticularis

The oothecae of G. *lenticularis* collected in the field were usually attached to flat substrates such as long stemmed grasses or sticks. The oothecae are not oval or covered with the usual foamy sheath as with several other species in the Mantidae family, i.e., *Stagmatoptera supplicaria* Burmeister 1838, as depicted by [23]. *Galepsus lenticularis* oothecae are oblong in form, dorsally flattened and usually light to dark brown in color. Hatched oothecae can be identified by the presence of white eclosion sack-like structures present on the greyish dorsally-flattened area of emergence. Measurements of ootheca parameters were done as indicated in Figure 1c. The ootheca length was measured as the area of emergence and did not include the residual process. To determine the number of eggs per ootheca, oothecae were dorsally dissected along the length and inspected under a microscope. The residual process was also dissected but did not contain any egg chambers. Eggs were arranged in oblong rows of between 2 and 3 eggs each, arranged next to each other (Figure 1c).

A total 42 oothecae were produced by the 18 captive reared and 9 field collected *G. lenticularis* females. Nine of these 42 oothecae were fertilized and hatched and were produced by the 9 field-collected females that were bred with field collected males under captive breeding conditions. The field-collected females produced 19 oothecae which never hatched (unhatched). Fourteen unfertilized oothecae were laid by 18 unmated females in the terrariums and thus no nymphs emerged from these unfertilized oothecae. No breeding with the captive-reared females were done because the possibility of parthenogenesis was also investigated, which has been recorded in other mantid species, i.e., *Coptopteryx viridis* Giglio-Tos 1915 (Coptopterygidea) [25], *Miomantis paykulli* Stäl 1871 [26], and in the Springbok mantis, *Miomantis caffra* Saussure, 1871 (Mantidae) [27]. Only nine of the 18 unmated captive-reared females produced oothecae during their lifecycle. Five of these females each laid two unfertilized oothecae. The pre-oviposition period in the case of unfertilized ootheca was 53 days (mean female age of 214 days). The period between laying of the two unfertilized oothecae was 20 days (mean female age of 235 days). The longest that a female lived after laying a final unfertilized ootheca was 50 days (mean female age of 285 days).

The length of the oothecae ranged between 18.9 and 30.0 mm (Figure 1c). The numbers of eggs per ootheca varied between the different types of oothecae. Unfertilized oothecae contained a mean of 36.6 eggs while the hatched and unhatched oothecae contained 50.2 and 59.2 eggs per ootheca, respectively (Table 3).

No abnormalities or noticeable morphological differences were observed between hatched, unhatched and unfertilized oothecae (Table 3). Unfertilized oothecae were significantly (*p* = 0.0033) shorter (11.10 mm) than those that hatched (*p* = 0.0005) and 7.40 mm shorter than the unhatched (*p* = 0.0059) oothecae. The number of eggs per ootheca was significantly (*p* = 0.0068) higher in unfertilized than unhatched field-collected ootheca (*p* = 0.0048). Despite the significant differences in length between the three oothecae types, no statistical difference in the width or height were recorded (Table 4).

### 3.3. Developmental Parameters

The mean overall hatch rate was 40.3%. Of the 192 neonate nymphs that hatched, 76 reached the 2nd instar and 48 (63%) of these completed their entire lifecycle. Eleven (40.0%) of the individuals that hatched reached adulthood (Table 5). The mean duration from hatch to adulthood was 21 weeks (148 days) (Table 5). While nymphs mostly became adults after seven molts some exceptions were recorded. Six individuals required ten molts to reach adulthood (three males and three females) and is therefore included in Table 5. One male became an adult after only four molts.

Although no statistical differences were found between the male and female development times or the duration of an instar per sex, a difference (*p* = 0.00001) was recorded between adult longevity of females and males (Table 5). The mean longevity (first instar to death) of females was 253 days while male longevity was 167 days and females and males lived for 93 and 26 days respectively after reaching adulthood (Table 5). The mean duration per instar was largely similar for males and females (Table 5).

The sex ratios of nymphs differed between individual oothecae but were predominantly male biased. Overall, 57% of the nymphs that survived to adulthood were males and 43% were females (Table 6). However, of the 48 individuals that reached adulthood, 18 (37.5%) were female and 30 (62.5%) males. 

## 4. Discussion

### 4.1. Distribution Patterns of Galepsus spp. in Southern Africa

The distribution records of Mantodea in general are widely dispersed with various records also in the MNHN (France), United States National Museum, The Natural History museum (London) and various German institutes [28,29,30,31]. The lack of taxonomic expertise concerning Mantodea which exists within Africa requires that specimens collected in the region be identified by experts outside the continent. 

Only six records of male *Galepsus* specimens were collected in Africa [31], one record of *G. capitatus* and five of *G.* (*Onychogalepsus*) *meridionalis* (Saussure, 1872) var. *montana* males from Kenya. These two species as well as the eight other species were recorded throughout South Africa [10,13]. However, four *Galepsus* species, i.e., *G.* (*Onychogalepsus*) *focki* Werner 1923, *G.* (*Lygdamia*) *brincki* Beier 1955, *G.* (*Onychogalepsus*) *ulricae* Kaltenbach 1996, and *G.* (*Onychogalepsus*) *letabaensis* Kaltenbach 1996, were listed to occur in Southern Africa [10,13]; however, no records of these species were found in any of the insect collections visited during this study. No specimen records of *G. centralis* occurs in the collections in South Africa, which includes the list compiled by Kaltenbach [10]. However, only two specimens of *G. centralis* were previously collected, one in Tanzania and another in the Democratic Republic of the Congo [2,5]. These two records were also those used for the original species description of *G. centralis* by Beier in 1957 [32].

It should be noted however that no consistency with regards to sampling methods exist for museum records. The distribution map (Figure 2) indicates that the distribution of *Galepsus* in Southern Africa is associated with the grassland and savanna biomes. This could explain the lack of records from the Cape Floristic region which is one of the most biological diverse areas in South Africa [33,34].

The subgenus *Onychogalepsus* seems to occur predominantly in grassland and savannah in South Africa while the *Lygdamia* subgenus seems to be widely distributed throughout Southern Africa (Table 1 and Figure 2).

Interestingly, the single record of *G. bipunctatus* and only representative of the subgenus *Syngalepsus* was collected at Pafuri, in the Limpopo province of South Africa. It was noted that the only specimen (*G. bucheti*) collected during their expedition in Central African Republic which belongs to the subgenus *Syngalepsus* was collected by means of a light trap on the banks of the Sangha river with “Arboreal stratum” [16]. The habitat depicted by means of photographs in Moulin [16] is similar to that of Pafuri with a similarly large river (Limpopo River) and tree-dominated vegetation. This could be an indication of the habitat preferred by the subgenus *Syngalepsus*. Further investigation is required to shed light on the habitat of this species. 

*Galepsus* records in South Africa indicated that more specimens where collected outside of protected areas. This may suggest that *Galepsus* exists in areas that are subject to disturbances, which could indicate that *Galepsus* is either a common species, or that it is highly adaptable. Protected areas are ideal study sites to generate baseline biodiversity data since they are often rich in biodiversity and are important for a wide range of species, due to these areas being viewed as “natural and pristine” without major disturbances [35,36,37,38,39]. A possible explanation for the collection bias is the higher population density and collection activities associated with these areas. The higher numbers of records outside of protected areas is most likely due to the ease of collecting in these areas, compared to in protected areas, especially for amateur collectors. The red tape associated with acquiring of permits for collection in protected areas makes the practice of collecting of specimens by citizen-scientists virtually impossible.

Old records or museum data can contribute to establishing baseline data regarding biodiversity within a region [40]. Historical data are also a source of distribution records and potential biodiversity and ecological information [41]. Battiston et al. [28] indicated that old records and descriptions were important with regards to the ecology of mantids in the Mediterranean area, and since little was known about mantids in this region, old records were used in highlighting conservation issues for Moroccan mantids. For example, mantid specimens in museum collections in Morocco was collected 78 years prior to the investigation [28], and when the locality description information of *Tenodera rungsi* Uvarov 1935 was revisited in 2011, a population of *T*. *rungsi* was still present at the described locality [28]. The occurrences and distribution of the genus *Galepsus* in this study is based on museum collection records and is another example of the validity and importance of museum collection records. 

### 4.2. Biology of Galepsus Lenticularis

There is a lack of literature about not only the biology of *Galepsus* spp., but the entire Tarachodidae family. A study by Ene [42] on *Tarachodes* (*Barbachodes*) *afzelii* Stäl 1871, which occurs in west Africa, is the only other study with which the data of this study on the biology of *G. lenticularis* can be compared to. The recent rearrangement of the systematics of the entire Mantodea order [9] also now places *Galepsus* and *Tarachodes* in the same subfamily (Tarachodinae), thus validating comparisons to results obtained by Ene [42]. The oothecae of this other species in the Tarachodidae family, *T. afzelii,* is constructed in a simplistic and unordinary manner, which differs from the majority of Mantodea oothecae [42]. The latter description of the oothecae is quite similar to that of *G. lenticularis*. The construction of more “primitive” oothecae by *G. lenticularis* and *T. afzelii* closely resembles that of Blattodea oothecae [42,43]. This is in accordance with the phylogenetic position of *Galepsus* [44]. It was suggested that the lack of the characteristic protective air-filled and foamy sheath coating of the oothecae might be the reason that females of *T. afzelii* exhibit a degree of parental care and guard oothecae during the incubation period and up to 48 hours after nymphs hatched [42]. However, no such behaviour was noted during this study on *G. lenticularis.*

The size of the oothecae of *Galepsus* and *Tarachodes* was similar (24.7 mm in length for *G. lenticularis* and 30.0 mm for *T. afzelii*). However, the mean number of eggs per ootheca was 50 and 129 for *G. lenticularis* and *T. afzelii* respectively. Despite this difference in number of eggs per oothecae, it was indicated that field-collected oothecae and oothecae obtained from laboratory studies of *Orthodera ministralis* Fabricius 1775 (Mantodea: Mantidae) did not differ significantly in structure [45]. 

Various aspects such as temperature, food, water limitations and rainfall have been shown to influence ootheca structure [42,45,46,47]. However, the difference observed in *G. lenticularis* oothecae (unhatched and hatched and unfertilized) with regard to the number of eggs and length of the oothecae could indicate that these females would rather conserve valuable resources, instead of producing unfertilized oothecae which do not produce offspring. In contrast to this, a recent study [48] indicated that the length of the ootheca of a cockroach species (*Periplaneta americana*) Linnaeus 1758 (Blattodea: Blattidae), was not influenced by the fertility of the oothecae. Although *P. americana* can also reproduce through parthenogenesis, it is highly likely that females would not invest valuable resources into formation of oothecae if it produces no genetically diverse offspring. No parthenogenesis was recorded for *G. lenticularis* in this study, despite it being recorded for some other mantid species [26,49,50].

The mean incubation period of *G. lenticularis* oothecae was 20 days (Table 5). Similar incubation periods were recorded for *T. afzelii*, with incubation periods ranging between 18 and 21 days under field conditions and 25 days under laboratory conditions [42]. The duration of the incubation period of eggs of *Tenodera aridifolia aridifolia* Stoll 1813 (Mantodea: Mantidae) was between 14 and 21 days at 30 °C [51]. Higher temperatures have been indicted to result in shorter incubation periods [42,45]. Recently, it was documented that the incubation period of *Ephestiasula rogenhoferi rogenhoferi* Saussure 1872 (Hymenopodidae) (previously known as *Ephestiasula pictipes* Wood-Mason 1879) is between 15.2 and 16.9 days during various seasons i.e., late winter–summer, monsoon and post monsoon–early winter (2013–2014) [52]. The interval between laying of the two unfertilized oothecae by *G. lenticularis* were laid at an interval of was 20 days, while this interval was 36 days for *T. afzelii* [42].

### 4.3. Developmental Parameters

No significant differences (*p* = 0.09) (Table 5 and Table 6) in male and female nymphal developmental periods were recorded. Similarly, no significant differences were reported to exist between the nymphal development periods of *E. rogenhoferi* [52] and *T. afzelii* [42]. The adult females of *G. lenticularis* lived nearly three times as long (91 days) as the males (26 days). Similarly, it was found that female *T. afzelii* lived twice as long as males, irrespective of whether the female was mated or not [42]. Although food limitations may influence adult longevity, a case was recorded where an adult female lived for 44 days without food [42]. Female longevity was also significantly longer than that of males under both laboratory and field conditions for *Iris oratoria* (Linnaeus 1758) (Mantodea: Tarachodidae) [53]. *Ephestiasula rogenhoferi* female adult longevity was 20 to 25 days longer than that of males [52].

It has been suggested that the longevity of adult females of *I. oratoria* was the reason that the sex ratio of this species changed over time after spring commences [52]. While the sex ratio during the 1st-instar is 1:1, it changed due to comparatively higher mortalities amongst male individuals over time, resulting in the sex ratio becoming female-biased later in the season. However, in this study, under captive breeding and laboratory conditions, the sex ratio for *G. lenticularis* was male dominated. Some variation did exist but in seven of nine of the ootheca that hatched, the sex ratio favored males. A similar change in sex ratio over time was reported for a *Tenodera sinensis* Saussure 1871 (Mantidae) population in the USA, where the population was male dominated during one year, but not the following year [54]. This could indicate that fluctuations in sex ratios of mantid species can occur between years. 

Since female *G. lenticularis* cannot fly, a male dominated population is required as males need to find females to mate. Males, due to their flight capability, are more likely to be subject to predation by bats and birds [42,55,56]. Although cannibalism was not recorded for *G. lenticularis* during this study, cannibalism might occur in nature if females are not as well fed as they were during this study. Another hindrance to male mantids are that they tend to be more attracted to light which is a factor which could increase the likelihood of them becoming prey [57,58]. Males of *T. sinensis* have a larger home range size (55.05 m^2^) in comparison to the females (23.78 m^2^) [54], which increases the opportunity for multiple matings to occur.

The variance in number of nymphal instars, duration of the stages, as well as female adult longevity and reproductive capability of *G. lenticularis* could be strategy to reduce competition between siblings for limited resource. First instar nymphs require approximately 14 days to develop to the second instar. Similarly, the period between production of the 1st and 2nd ootheca by *G. lenticularis* females was approximately 20 days, which is also the incubation period of an ootheca. This would allow first instar nymphs to become second instars before the younger ootheca hatches, which would then decrease the likelihood of a particular female’s genetic progeny to compete for resources. Although no female was recorded producing more than two oothecae in this study, *T. afzelii* were recorded to produce up to five oothecae per female [42]. 

Phenological differences in oothecae have been observed for *Tenodera sinensis* Saussure 1871 and *Tenodera angustipennis* Saussure 1869 [59,60] and also between *T. angustipennis* and *Tenodera aridifolia* [61]. Hurd and Eisenberg [59] suggested that the differences in the periods to hatching of oothecae were an evolutionary adaptation to mitigate inter-guild competition between nymphs of different species. The nymphs occur in the same habitat and stratum and therefore compete for limited food resources [59]. The long period between oothecae production by *G. lenticularis* females could therefore also be a strategy to reduce resource competition between siblings, or inter-guild competition with other grassland mantid species. It was suggested that temporal differences in oviposition could be an evolutionary advantage for certain species [62]. For example, oothecae of *T. angustipennis*, which is a small species, are laid much later in the season, compared to that of the bigger *T. sinensis,* which feed on later-hatching and smaller individuals of *T. angustipennis*.

Christensen and Brown [54] reported that the abundance of *T. sinensis*, a mantid species that occurs in the State of New York (USA), ranged between 10 and 39 mantids per 1000 m^2^ and that females with larger abdomens (presumably ready to lay an ootheca), would travel greater distances than non-gravid females. Female activity and movement could therefore also be influenced by the availability of suitable substrates and micro-habitats on which to attach oothecae. This possible increase in movement, along with the above-mentioned synchronicity of incubation periods of oothecae as well as nymphal developmental stages, could further increase the survival rate of the progeny of a particular *G. lenticularis* female by decreasing sibling resource rivalry or competition. Further investigation into movement patterns and density of field populations of *G. lenticularis* could shed some light on this proposed survival strategy. The hatch and survival rate of 40% and 25% respectively, recorded for *G. lenticularis* in this study would most likely be much lower under field conditions, which may lead to further decreases in competition among siblings.

There were large variations in developmental parameters of nymphs that emerged from a single ootheca, similar to what was reported for *Stagmomantis limbata* Hahn 1835 (Mantidae) [63]. Under field conditions this could be as a result of multiple paternities [51]. However, in this study, females were limited to breeding with one male to prevent multiple paternities, but field collected females could have been inseminated by more than one male before their oothecae were collected. 

Multiple paternities have been indicated to be possible in *T. aridifolia* [51], and it was suggested that multiple sperm storage organs could be a strategy use by female arthropods to control their paternity [64]. A study reported the differences in the number of male parents per ootheca in two mantid species of the Liturgusidae family, i.e., *Ciulfina rentzi* Holwell, Ginn and Herberstein 2007 and *Ciulfina klassi* Holwell, Ginn, and Herberstein 2007 [65]. In the latter example, between four and six male parents contributed to a single ootheca of *C. klassi*, while only one male parent was responsible for a single ootheca of *C. rentzi* [66]. The production of oothecae with multiple paternities could theoretically be possible and could increase genetic diversity within a localized population, which could also increase survival of a species such as *G. lenticularis*. However, further research regarding the possibility of multiple paternities in the *G. lenticularis* should be investigated to determine this theoretical possibility. 

## 5. Conclusions

*Galepsus* is widespread in Southern Africa and it seems more prevalent in grassland and savanna areas. The presence of only single specimens of *G. bipunctatus* Beier 1931 and *G.* (*Onychogalepsus*) *centralis* Beier 1957 in the museum collections in South Africa could indicate that it is possibly rare and that conservation thereof is required. This may however also be a by-product of the lack of sampling and taxonomic expertise. This study is the first to describe the biology of *G. lenticularis* and distribution *Galepsus* in Southern Africa and highlights the importance of museum collections. Museum collections have large numbers of records that contain distribution data, which will become more important because it enables the identification of possible habitable and favorable areas for species of which little is known, for example *Galepsus* in Southern Africa. 

## Figures and Tables

**Table 1:** The eleven recorded *Galepsus* species throughout Southern Africa and their associated taxonomic nomenclature.

Family	Subgenus	Species
Tarachodidae	*Syngalepsus* Beier, 1954	*Galepsus bipunctatus* Beier, 1931
Tarachodidae	*Onychogalepsus* Beier, 1954	*Galepsus capitatus* Saussure, 1869
Tarachodidae	*Onychogalepsus* Beier, 1954	*Galepsus centralis* Beier,1957
Tarachodidae	*Onychogalepsus* Beier, 1954	*Galepsus damaranus* Giglio-Tos, 1911
Tarachodidae	*Onychogalepsus* Beier, 1954	*Galepsus femoratus* Giglio-Tos, 1911
Tarachodidae	*Onychogalepsus* Beier, 1954	*Galepsus intermedius* Werner, 1907
Tarachodidae	*Lygdamia* Stäl, 1877	*Galepsus lenticularis* Saussure, 1872
Tarachodidae	*Onychogalepsus* Beier, 1954	*Galepsus meridionalis* Saussure, 1872
Tarachodidae	*Onychogalepsus* Beier, 1954	*Galepsus pentheri* Giglio-Tos, 1911
Tarachodidae	*Onychogalepsus* Beier, 1954	*Galepsus rhodesicus* Beier, 1954
Tarachodidae	*Onychogalepsus* Beier, 1954	*Galepsus transvaalensis* Beier, 1954

**Table 2:** Comparison of the number of specimen records for each *Galepsus* species and thus its distribution throughout Southern Africa as recorded throughout the museums of South Africa. It should be noted that 285 specimens that were recorded in the museum collections of South Africa were undefined (labelled as 165 G. sp.).

Countries in Southern Africa									
Species	Angola	Bots- wana	Lesotho	Mozam- bique	Namibia	South Africa	Eswatini	Zim- babwe	Zambia
*Galepsus* sp.	12	5	1	6	25	285	1	14	-
*G. bipunctatus*	-	-	-	-	-	1	-	-	-
*G. capitatus*	-	-	-	-	-	9	-	4	-
*G. centralis*						1			
*G. damarnus*	-	1	-	-	-	-	-	-	-
*G. femoratus*	-	-	-	-	1	2	-	1	-
*G. intermedius*	-	-	-	1	-	24	-	-	1
*G. lenticularis*	1	-	-	1	5	10	-	1	-
*G. meridionalis*	-	-	-	1	1	5	-	1	-
*G. pentheri*	-	-	-	-	-	2	-	1	-
*G. rhodesicus*	-	-	-	-	-	-	-	-	1
*G. transvaalensis*	-	-	-	-	-	11	-	-	-

**Table 3:** Mean size and number of internal egg chambers of the various types of oothecae of *Galepsus lenticularis* reared under captive breeding conditions. SD = Standard deviation.

Oothecae (42)	Length (cm) ± SD	Width (cm) ± SD	Height (cm) ± SD	Number of eggs ± SD
Overall (42)	2.47 ± 0.76	0.24 ± 0.059	0.31 ± 0.070	49.79 ± 21.12
Unfertilized (14)	1.89 ± 0.44	0.26 ± 0.063	0.30 ± 0.068	36.64 ± 15.35
Unhatched (19)	2.63 ± 0.76	0.22 ± 0.053	0.30 ± 0.074	59.26 ± 22.52
Hatched (9)	3.00 ± 0.61	0.25 ± 0.050	0.30 ± 0.070	50.20 ± 15.74

**Table 4:** Analysis of variance (ANOVA) and associated post hoc Tukey p-values between the three types of ootheca and the various morphological parameters.

Statistical Test	Oothecae	Length	Width	Height	Number of Eggs
**ANOVA**	Overall	0.0033 *	0.0503	0.8390	0.0068 *
**Post Hoc** **(HSD Tukey)**	Unfertilized × Unhatched	0.0059 *	0.0477 *	0.8608	0.0048 *
	Unhatched × Hatched	0.2753	0.2979	0.8926	0.4765
	Hatched × Unfertilized	0.0005 *	0.8227	1.000	0.2304

Significant *p*-value < 0.05, indicated by *.

**Table 5:** Mean duration (in days) of each of the respective life stages of *Galepsus lenticularis* and differences between male and female development under laboratory conditions.

	Mean Duration (days ± SD)			
Life Stage	Overall	Males	Females	*p*-Value
Ootheca (incubation period)	20.25 ± 6.3	19.16 ± 4.94	22.06 ± 7.91	0.125
1st Instar	14.39 ± 3.91	14.80 ± 4.60	13.72 ± 2.32	0.361
2nd Instar	15.77 ± 10.57	15.70 ± 11.60	15.88 ± 8.91	0.953
3^rd^ Instar	18.38 ± 11.04	20.33 ± 13.03	15.11 ± 5.43	0.114
4th Instar	23.22 ± 15.05	21.70 ± 13.10	25.77 ± 17.95	0.369
5th Instar	27.97 ± 21.78	24.13 ± 13.10	34.16 ± 29.81	0.126
6th Instar	26.02 ± 12.46	25.03 ± 12.95	27.58 ± 11.85	0.515
7th Instar	23.78 ± 15.95	23.42 ± 16.03	24.30 ± 16.46	0.880
8th Instar	19.22 ± 6.66	17.81 ± 5.25	21.42 ± 8.40	0.275
9th Instar	22.16 ± 13.34	14.33 ± 10.69	30.00± 12.12	0.169
Total nymphal period *	148.85 ± 40.44	141.20 ± 36.06	161.61 ± 45.03	0.091
Adult longevity **	50.66 ± 40.02	26.30 ± 15.44	91.27 ± 35.03	0.000*
Pre-oviposition period	53.00 ± 26.50	N/A	53.00 ± 26.50	N/A
Interval between oothecae	20.00 ± 14.10	N/A	20.00 ± 14.10	N/A
Period from hatch to death	199.16 ± 61.31	166.93 ± 38.79	252.88 ± 54.20	0.000*

* From ootheca hatch to final molt (1st Instar – 8th/9th instar). ** Duration of adult phase.

**Table 6:** The mean hatch rate, survival rate and gender dynamics throughout the study that resulted from each individual field-collected female kept in the laboratory and each produced a single fertile ootheca.

Ootheca No.	No. of Days from Oviposition to Nymph Emergence	No. of Eggs per ootheca	Fertility (%)	Survival (%)	Male(%)	Female (%)	Sex Ratio (♂:♀)
Ootheca 1	41	76	67.11	05.88	33.33	66.67	1:2
Ootheca 2	11	34	08.82	33.33	100.0	00.00	1:0
Ootheca 3	17	66	13.64	11.11	00.00	100.0	0:1
Ootheca 4	16	49	32.65	18.75	66.67	33.33	2:1
Ootheca 5	18	45	68.89	80.65	68.00	32.00	2:1
Ootheca 6	20	64	54.69	08.57	33.33	66.67	2:1
Ootheca 7	21	49	30.61	06.67	100.0	00.00	1:0
Ootheca 8	19	41	60.98	32.00	75.00	25.00	3:1
Ootheca 9	14	28	25.00	42.86	33.33	66.67	1:2
Mean ± (SD)	19.7± 8.6	50.2 ± 15.7	40.3 ± 23.1	25.6 ± 24.3	56.6 ± 33.9	43.4 ± 33.9	1.6:1
